# Effectiveness of a Program Intervention with Reduced-Iron Multiple Micronutrient Powders on Iron Status, Morbidity and Growth in Young Children in Ethiopia

**DOI:** 10.3390/nu10101508

**Published:** 2018-10-15

**Authors:** Aregash Samuel, Inge D. Brouwer, Edith J. M. Feskens, Abdulaziz Adish, Amha Kebede, Luz Maria De-Regil, Saskia J. M. Osendarp

**Affiliations:** 1 Ethiopian Public Health Institute, Gulele Sub City, Addis Ababa, Ethiopia; aregash.hafebo@wur.nl (A.S.); amha.kebede@gmail.com (A.K.); 2 Division of Human Nutrition and Health, Wageningen University, 6700 AA Wageningen, The Netherlands; edith.feskens@wur.nl (E.J.M.F.); saskia.osendarp@osendarpnutrition.com (S.J.M.O.); 3 Nutrition International Ethiopia, Nifas Silk Lafto Sub City, Kebele 04, Addis Ababa, Ethiopia; aadish@nutritionintl.org; 4 Nutrition International Headquarters, Ottawa, ON K2P2K3, Canada; lderegil@nutritionintl.org

**Keywords:** micronutrient powders, program effectiveness, infant and young child feeding, morbidity, growth

## Abstract

Despite the potential for improving iron status and child growth in low- and middle-income settings, concerns on the safety of high iron dosages of Micronutrient Powders (MNP currently limit their applicability in programs. We examined the effectiveness and risks of an integrated complementary feeding program with low iron dose (6 mg/serving) MNP among 6–23-month-old Ethiopian children using a quasi-experimental study design comparing children from five intervention districts (*n* = 1172) to those from four matched non-intervention districts (*n* = 1137). Haemoglobin concentrations increased in intervention and decreased in non-intervention children (group-difference +3.17 g/L), but without improvement in iron stores. Intervention children were 2.31 times more likely to have diarrhoea and 2.08 times more likely to have common cold and flu, but these differences decreased towards the end of the intervention. At end line, intervention children had higher mean Height-for-Age Zscore (HAZ) and a 51% reduced odds of being stunted compared to non-intervention children. MNP with low iron dose, when provided combined with other Infant and Young Child Feeding (IYCF) interventions, marginally improved haemoglobin status and resulted in a remarkable improvement in linear growth in 6–23-month-old children. These benefits likely outweigh the relatively small increase in the risk of diarrhoea.

## 1. Introduction

Micronutrient deficiencies are a global health burden, especially for young children in developing countries, because of poor quality diets and frequent infectious diseases. The high prevalence of deficiencies and their important adverse consequences on mortality, morbidity and disability result in a substantial disease burden. In particular, deficiencies of vitamin A and zinc increase the risk of child mortality, and zinc deficiency increases infectious morbidity and reduces linear growth as well. Deficiencies of iodine and iron are significant primarily for their effects on development and cognition and consequent disabilities [1,2]. Overall, it has been estimated that micronutrient deficiencies account for about 7.3% of the global burden of disease, with iron and vitamin A deficiency ranking among the 15 leading causes of the global disease burden [3]. The World Health Organisation (WHO) recommends point-of-use fortification of foods with iron-containing multiple micronutrient powders (MNP) for young children in populations with a prevalence of childhood anaemia greater than 20% [4]. Home fortification of foods with powders containing multiple micronutrients is recommended as an alternative to increase the vitamin and mineral intake in children 6–23 months of age because micronutrient deficiencies often co-exist [1].

Home fortification with iron-containing MNP has been shown to reduce both anaemia and iron deficiency anaemia (IDA) in infants and young children [5]. Because of its potential to reduce anaemia and improve micronutrient intakes, MNP are increasingly being used in Infant and Young Child Feeding (IYCF) programs in low- and middle-income countries. However, concerns around the safety of iron-supplement interventions among iron-replete children in both malaria-endemic and malaria-free areas have arisen, as the daily provision of supplemental doses of iron may exacerbate the presence and severity of infections, including malaria and diarrhoea [6,7,8]. The observed increased risk of infectious diseases may have been caused in part by an increase in pathogenic bacteria in the gut due to unabsorbed iron [8]. These studies have invigorated the debate around the safety of (daily) iron interventions, particularly in very young children, with adequate iron status, who may not yet have the capacity to adequately regulate iron absorption. In addition, in Ethiopia, with 34% of 6–59-month-old children having anaemia and 9% having IDA in 2016, the high iron intakes found in the Ethiopian national food consumption survey of 2013 have raised questions regarding necessity and safety of additional iron interventions. A low-dose iron supplementation may therefore be preferred to reduce side effects [9], but it is unclear to what level the daily iron dose in MNP should be reduced to retain efficacy against anaemia and at the same time reduce the adverse impact on morbidity.

Previous studies conducted in Ghana, Cambodia and Bangladesh, with similar settings and infection rates as in Ethiopia, used multiple micronutrient supplements including 12.5 mg of elemental iron per serving, among children aged 6–18 months [7,8,9,10,11,12,13]. In these settings, daily use of MNP with iron did not result in an increased incidence of malaria or other infectious diseases and was effective for preventing or treating anaemia. Lower dosages (6 mg iron per daily serving) were found to be efficacious in improving iron status in a trial involving small-quantity Lipid-Based Supplements (sq-LNS, 20 g lipid-based spreads to be used as a home fortificant) in Burkina Faso without increase in incidence of diarrhoea and malaria [14]. None of these studies, however, evaluated the use of a lower dose iron MNP in a scaled-up program context with limited control over intake and compliance.

We assessed the effectiveness as well as risks and benefits of a low-dose iron MNP (6 mg iron per serving every other day) on iron status, morbidity and growth of Ethiopian infants and young children within the context of a program on local complementary food ( CF) production.

## 2. Materials and Methods

### 2.1. Study Design and Participants

Within the Ethiopian community based nutrition program, the organisation Nutrition International (NI), together with the United Nations Children’s Fund (UNICEF) and implementing partners, implemented a local CF production (Grain Bank) program to improve the quality of CF and IYCF practices in four regions (Amhara, Oromia, Tigray and Southern Nations, Nationalities and Peoples’ Region (SNNPR) in Ethiopia [15]. Our MNP study was conducted in two of the four program regions, Oromia and SNNPR. We employed a quasi-experimental matched-control design in which outcomes were compared between children of intervention and non-intervention villages (kebeles). In SNNPR, 3 out of 5 and in Oromia 2 out of 5 Grain Bank program districts (woredas) were included as intervention districts. Per intervention district, 3–6 villages were selected based on status of grain bank in the village, number of children below 12 months of age and accessibility during rainy season. These so-called intervention villages (*n* = 17) were matched with 18 non-intervention villages in a 1:1 ratio. In one district, one large intervention village was matched with 2 non-intervention villages to equal the population size. Matching was done based on pre-set criteria including similar geographical and ecological conditions, existence of other health or nutrition programs, and livelihood data.

Primary outcomes were haemoglobin (Hb), anaemia and diarrhoea. Secondary outcomes were other iron status indicators (serum ferritin (SF), iron deficiency (ID), and iron deficiency anaemia (IDA)), morbidity of other diseases (common cold and flu, and measured fever) and growth including Height-for-Age Z-score (HAZ), Weight-for-Height Z-score (WHZ), Weight-for-Age Z-score (WAZ), and Height-for-Age Difference (HAD).

The sample size was calculated based on Hayes and Bennett [16]. We assumed a 11.7% incidence of diarrhoea in children 0–2 years of age among children in the non-intervention area [17]. We expected a 12% increase in the incidence of overall diarrhoea in the intervention group (assumed incidence of 5.3 episodes per year per child). This assumed effect was based on the observed Incidence Rate Ratio (IRR) of 1.12 for overall diarrhoea after introduction of MNP in a large effectiveness study in Pakistan [6], as this was the largest study to date to observe an adverse effect on diarrhoea after MNP supplementation. A sample size of 1170 per group would enable us to detect this increase of incidence of overall diarrhoea with 80% power and two-sided *p*-value of 5% with an assumed drop-out rate of 10%.

For anaemia, we assumed a prevalence of 73% in 6–11-month-old Ethiopian children based on 2012 data from the Ethiopia Demographic and Health Survey, and expected to observe a 30% decrease in anaemia concentration, which was the average reduction in anaemia prevalence observed in a systematic review of studies with iron-containing multiple micronutrient powders [5]. Based on a power of 80%, a two-sided *p*-value of 5%, a 10% drop-out rate and design effect of two for the matched-controlled design, we aimed to collect biochemical data on 130 children in the control and 130 in the intervention group.

**Figure 1:**
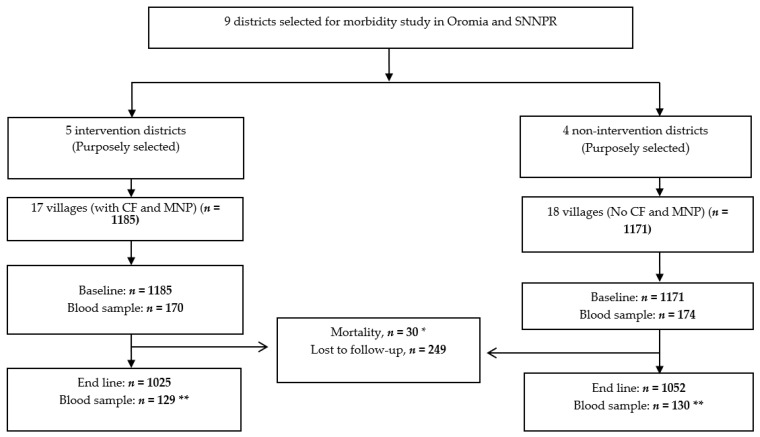
Study profile. * Thirty children died (five from intervention villages before the MNP intervention started and 25 from non-intervention villages throughout the study period). ** End line blood samples were taken from 259 of the 344 children with blood samples collected at baseline: 41 were absent, 1 was a severe-acute-malnutrition case, 2 were transferred and 41 refused to give a blood sample. SNNPR: Southern Nations, Nationalities and Peoples’ Region; CF: complementary food; MNP: multiple micronutrient powder.

Target children were aged 6–11 months at baseline to ensure that all children continued to be eligible to receive the program intervention for the entire duration of the follow-up. After listing all children below one year of age in each selected village by health extension workers, parents were informed and invited to bring their child for screening. Per village, about 65 eligible children were included in the study, resulting in a total of 2356 children, 1185 in the intervention group, which received low-dose iron MNP (30 sachets/two months) along with the CF from the Grain Bank program, and 1171 in the non-intervention group, which did not receive MNP and CF (Figure 1). The sub-sample for biochemical assessment was selected from these study children by going from study village to village, starting nearby the capital Addis Ababa, until the required number was obtained.

### 2.2. Data Collection Procedures

The study was conducted from March 2015 to May 2016, during which children were each followed for 52 weeks. Due to a delay in the delivery of MNP, children did not receive these during the first 15 weeks after end of baseline investigation, with a mean duration of this pre-intervention period of 93 ± 14.8 days. After this period, the MNP intervention was implemented for 37 weeks with a mean duration of intervention period of 182 ± 32.6 days.

The MNP intervention product MixMe^®^ was manufactured by DSM Nutritional Products in South Africa. Each sachet of MixMe^®^ contained 6 mg of iron as encapsulated ferrous fumarate, instead of the usual 12.5 mg iron, and 14 other essential vitamins and minerals (see Table 1).

The study team provided MNP at the local health post. Mothers/caregivers were given 30 sachets every 2 months and were instructed to administer 15 sachets per month to their children, one sachet every other day. Children were thus supposed to consume 6 mg of iron every other day. During monthly home visits, compliance and adherence were assessed by counting empty sachets. Overall, children consumed an average of 79% of the total servings of MNP received, during the intervention period.

Thirty-six well-trained data collectors and six field supervisors carried out data collection. Children who required medical treatment were referred to the nearest health facility. A data safety and monitoring board (DSMB) was constituted, consisting of a paediatrician, a physician and a public health scientist, all independent to the study. All adverse events were communicated to the DSMB within a maximum of two days.

Morbidity from infectious diseases such as diarrhoea, flu, and fever was assessed every two weeks by means of a pretested recall questionnaire. Children’s body temperature was measured every two weeks in the armpit using a digital thermometer with the precision of ±0.1 °C (SLC, TempCheck) and a child with a body temperature above 37.5 °C was referred to the health centre for malaria testing, further investigation and treatment.

Measurements of height, and weight were taken every quarter using standard procedures [18]. Height was measured on the UNICEF standard measuring board with a precision of 0.1 cm; weight was measured using UNICEF Seca 874 U electronic scales (UNICEF Supply Division, Copenhagen, Denmark) with 100 g precision, calibrated daily with a known weight. Children shorter than 85 cm were measured lying down, while those taller than or equal to 85 cm were measured standing up. Two measurements were taken. A third measurement was taken if the difference between the first two was more than 0.5 cm or 0.5 kg.

Iron status, haemoglobin (Hb), serum ferritin (SF), soluble transferrin receptor (sTfR), and inflammation markers, high sensitive C-reactive protein (CRP) and α1-acid glycoprotein (AGP), were measured at baseline and end line in a subsample of children. For this purpose, venous blood samples (3 mL trace element-free vacuettes and ethylenediaminetetraacetic acid (EDTA containing tubes) were collected at the health post of the study villages by highly skilled phlebotomists following the WHO blood collection protocol [19]. Samples were transported in cold boxes containing frozen gel packs (<−18 °C) to a nearby health centre or hospital for serum separation and aliquoting in cryovials. Serum samples were stored in deep freezers of the regional laboratories until transported to the Ethiopian Public Health Institute (EPHI) for storage at −80 °C and subsequent laboratory analyses after the final end line sample was obtained. Hb was analysed immediately (in the field) using a Hemocue^®^ photometer (Hb 301, Hemocue, Angelholm, Sweden). Hb concentration was corrected for altitude using Global Positioning System (GPS) data for the villages and altitude adjustment values as provided by the International Nutritional Anemia Consultative Group (INACG) [20]. Serum ferritin was analysed using Cobas e411 (Roche Diagnostics GmbH), a fully automatic run-oriented immunoassay analyser for the determination of immunological tests using the electrochemiluminescence immunoassay (ECLIA)process [21]. The concentrations of CRP, sTfR and AGP were analysed using Cobas 6000 (Roche Diagnostics, GmbH, Mannheim, Germany), using the immunoturbidimetric principle. The CV (inter-assay) for the various indicators were 6.7% for SF, 4.7% for CRP, 2.1% for sTfR, and 4.2% for AGP.

All questionnaires were manually checked for completeness before data entry in duplicate using CSPro 5.0 software (United States Census Bureau., Suitland, MD, USA).

### 2.3. Statistical Methods

Statistical analysis was done with SPSS version 22.0 (IBM Corporation, Armonk, New York, NY, USA). The analyses followed the intention to treat principle. Data distributions were checked by visual examination of Q-Q plots, histograms and tested for normality with the Kolmogorov–Smirnov test. Baseline characteristics of study children and their caregivers were summarized as mean (SD) for continuous variables which were normally distributed, or otherwise as median (25th and 75th percentiles), and as percentages for categorical variables. Descriptive data on intervention and non-intervention children were compared using the independent sample *t*-test for continuous variables, Mann–Whitney *U* test for skewed continuous variables and chi-square test for categorical variables.

For morbidity calculations, each randomized child contributed to the total number of observation days until they were lost to follow-up or until completion of the intervention. Fever was defined as body temperature >37.5 °C. The longitudinal prevalence of each illness, diarrhoea, common cold/flu, and fever, was calculated, dividing the total number of days being ill by the total days of observation per child, multiplied by 100. Additionally, we calculated the incidence rate per year for diarrhoea and common cold/flu as the number of sick cases per total number of children at the given time based on the study period and extrapolated to one year. This was based on the assumption that children would have experienced maximum one episode per recall period of 14 days. Morbidity was calculated during the pre-intervention period and intervention period separately. Differences in longitudinal prevalence of diarrhoea, common cold/flu, and measured fever were analysed with Generalized Linear Mixed Models (GLMM) adjusted for baseline values, age, gender, and treatment group. The morbidity observed during the pre-intervention run-in period was used as a proxy for baseline morbidity. To adjust for the matched-controlled design of the study, a proxy for matched pairs of districts were included in the models as random effects. Differences in diarrhoea and common cold/flu prevalence over time were analysed with GLMM using prevalence per 2 weeks observation round as dependent variable, and adjusting for interaction with time, baseline values, age, gender, and treatment group. Differences in incidence from diarrhoea and flu, and number of clinical visits, were analysed with Poisson regression using the number of episodes or clinic visits as the dependent variable, baseline incidence (diarrhoea and common cold/flu), age at baseline, gender, matching pairs and treatment groups as covariates, and including the total number of observation days as an offset term.

Anaemia was defined as Hb < 110 g/L and ID was defined as SF concentration < 12 µg/L. IDA was defined as Hb < 110 g/L with SF < 12 µg/L [21]. Inflammation was defined as CRP > 5 mg/L and/or AGP > 1.0g/L [22]. The Biomarkers Reflecting Inflammation and Nutritional Determinants of Anemia (BRINDA) internal regression correction (IRC) approach was used to correct SF concentrations for inflammation, [22] using a separate regression coefficient for intervention and non-intervention groups. The effect of the intervention on iron status was tested for the subsample of children with baseline and end line data, using linear regression analyses with change in altitude adjusted Hb, SF adjusted for inflammation, or sTfR as the dependent variable, and age and gender as covariates.

Weight-for-age (WAZ), length/height-for-age (HAZ), and weight-for-height (WHZ) were determined using WHO Anthro-Plus software version 3.2.2 (World Health Organisation, Geneva, Switzerland) based on 2006 WHO reference population [18]. Height-for-age differences (HAD) in cm was calculated by subtracting the median height (sex and age specific based on the WHO 2006 growth standards) from the measured height of the child [23]. During baseline data cleaning, 25 children with WHZ *z*-scores < −3.01 were excluded as not meeting inclusion criteria and 1 child was excluded from further analysis because of implausible value for *z*-score (>9.0). Linear Mixed Models (LMM) were used to compare longitudinal results of anthropometry between intervention and non-intervention children. Subject-level random effects were introduced to account for individual growth trajectories. After comparing the Akaike information criterion (AIC) values for model selection, we used the autoregressive model (AR1) for repeated effects and Variance Components (VC) for random effects. Time-trend interactions were analysed in the Linear Mixed Models to assess whether differences between treatment groups changed over time.

Differences between groups in categorical variables at end line were compared using logistic regression adjusting for age, gender and baseline values for anaemia, ID, and IDA, and adjusting for age, gender, matching-pair and baseline values when studying stunting, wasting, and underweight.

Tests of significance were 2-tailed, and *p* < 0.05 was considered statistically significant.

Ethical approval was obtained from the Ethiopian National Research Ethics Review Committee (NRERC). Signed consent was obtained from caregivers of the study children before participation in the study. The study was registered at http://www.clinicaltrials.gov/ with clinical trials identifier of NCT02479815.

## 3. Results

Baseline data were available for 2309 children of age 6–11 months from 17 intervention (*n* = 1172) and 18 non-intervention (*n* = 1137) villages. For biomarker analysis, a subgroup of 129 children from each group was analysed. Socio-economic characteristics of mothers and children were similar at baseline (Table 2). A majority of the study children were still breastfed (>93%). Mean age of mothers was 25.3 ± 5.8 years in the intervention group and 25.7 ± 5.7 years in the non-intervention group. Half of the mothers were illiterate (about 45–50%); more than 90% of the households owned land.

### 3.1. Iron Status

At baseline, Hb concentration was lower in intervention (112.4 ± 12.6 g/L) compared to non-intervention children (115.0 ± 9.7 g/L) (Table 3). At the end of the intervention period, Hb levels were 114.8 ± 10.5 g/L in the intervention group and 114.2 ± 8.7 g/L in the non-intervention group. Hb concentrations increased in children in the intervention group (+2.4 ± 1.2 g/L), and slightly decreased in children in the non-intervention group (−0.8 ± 1.2 g/L) with a borderline significant difference in difference estimate of 3.2 g/L (SE 1.7 g/L, *p* = 0.056) between intervention and non-intervention group. In contrast, SF concentrations increased in the non-intervention group (+6.42 ± 1.48 μg/L) between baseline and end line, whereas they decreased in the intervention group (−2.10 ± 1.55 μg/L) resulting in a significant difference in difference estimate of −8.53 μg/L (SE 2.14 μg/L, *p* < 0.0001). sTfR concentrations decreased in both intervention and non-intervention groups, with no difference between groups (Table 3).

**Figure 2:**
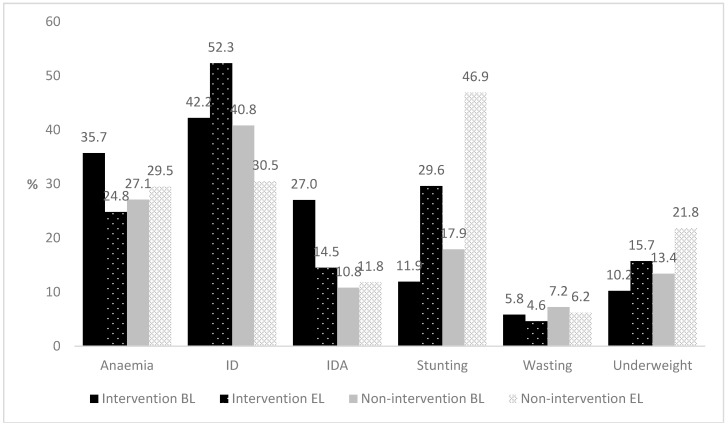
Prevalence of Anaemia, Iron Deficiency (ID), Iron Deficiency Anaemia (IDA), Stunting, Wasting and Underweight at baseline (BL) and end line (EL) in intervention and non-intervention groups. Differences in end line prevalence between intervention and non-intervention groups were tested with Logistic Regression, adjusted for age, gender, and baseline prevalence (and matched pairs for stunting, wasting and underweight).

The prevalence of anaemia and IDA reduced in intervention children, from 35.7% to 24.8% for anaemia and from 27.0% to 14.5% for IDA, while they both increased slightly in non-intervention children (Figure 2). Adjusting for baseline prevalence, the end line prevalence of anaemia (OR 0.76, 95% CI 0.44–1.33) and IDA (OR 1.09, 95% CI 0.49–2.43) were not significantly different between the treatment groups. In contrast, the prevalence of ID increased significantly in intervention children (from 42.4% to 52.3%) and decreased in non-intervention children (from 40.8% to 30.5%, OR for ID at end line: 11.3; 95% CI: 3.7–34.1).

### 3.2. Morbidity

**Figure 3:**
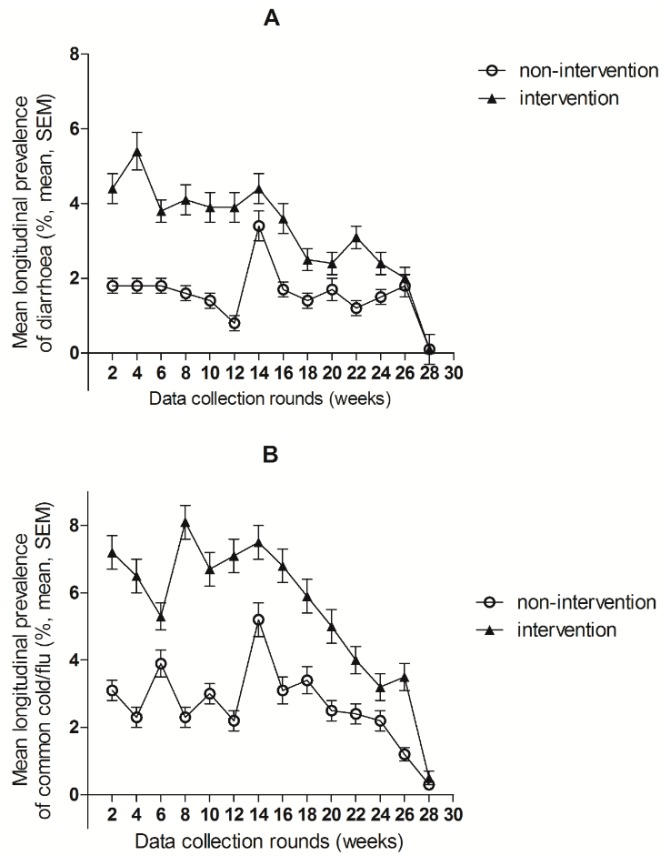
Mean longitudinal prevalence of diarrhoea (**A**) and common cold/flu (**B**) in intervention and non-intervention group over time at every 2 week measurement round. SEM = Standard Error of the Mean.

During the pre-intervention period, the mean (± SD) longitudinal prevalence of both diarrhoea (6.9 ± 6.8% vs. 4.9 ± 6.8%, β = 1.91, 95%CI: 1.38–2.45) and common cold and flu (8.0 ± 8.4% vs. 5.0 ± 8.7%, β = 2.98, 95%CI: 2.33–3.62) were significantly higher in the intervention compared to the non-intervention group. During the intervention period, we observed a significantly higher longitudinal prevalence of diarrhoea in the intervention group (2.7 ± 3.6%) compared to the non-intervention group (1.5 ± 3.2%, β = 1.01, 95%CI: 0.73–1.29) (Table 4). The average number of days of diarrhoea per episode was 4.7 ± 2.2 and 4.2 ± 1.8 (*p* < 0.001), in the intervention and non-intervention children respectively. Similarly, for common cold/flu, a significantly higher longitudinal prevalence (5.4 ± 5.4%) was observed in the intervention children compared to the non-intervention children (2.7 ± 3.7%, β = 2.44, 95%CI 2.08–2.80). There was no difference in longitudinal prevalence of (measured) fever between groups (β = −0.01, 95%CI: −0.03–0.01). During the intervention period, the incidence of diarrhoea was higher in the intervention compared to the non-intervention children (incidence rate ratio IRR: 2.31, CI 95%: 1.92–2.78). A higher incidence was also observed for common cold/flu (IRR: 1.43, CI 95%: 1.23–1.65). However, the incidence of clinic visits due to diarrhoea or common cold/flu were not different. The point prevalence at every two weeks visit for diarrhoea and common cold/flu in intervention and non-intervention children is shown in Figure 3. The differences between groups in point prevalence of diarrhoea and common cold and flu decreased over time (*p* < 0.001 for interaction with time).

### 3.3. Growth

**Figure 4:**
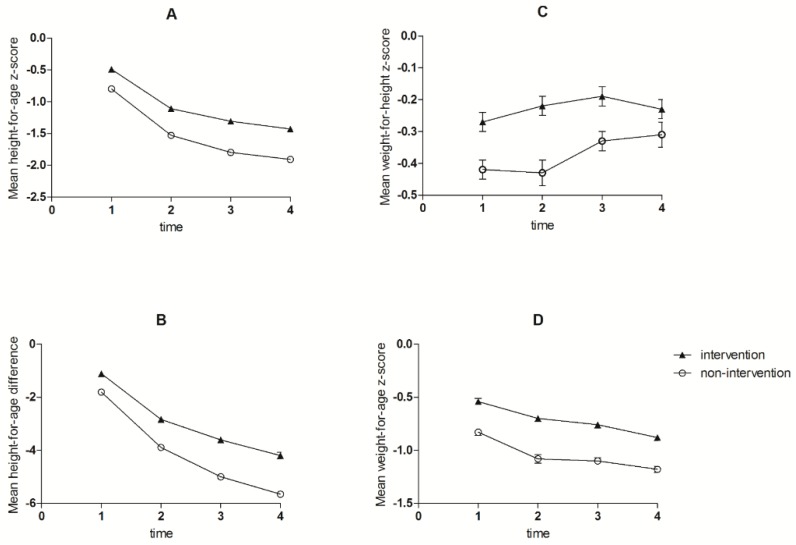
Mean height-for-age z-score (**A**); height-for-age difference (**B**); weight-for-height z-score (**C**) and weight-for-age z = score (**D**) of intervention during the study period.

Over the course of the intervention, children in the intervention group had a significantly higher length and weight gain than children in the non-intervention group (Table 5). At end line, mean HAZ was higher in intervention children compared to non-intervention children (adjusted β for difference in difference estimate: 0.18, SE: 0.05, *p* < 0.005, Table 5), and similar results were observed for HAD (β = 0.78, SE: 0.12, *p* < 0.005). No differences in end line WAZ (β = 0.01, SE: 0.04, *p* = 0.78) and WHZ (β = −0.09, SE: 0.05, *p* = 0.052) were observed. The changes in HAZ and HAD between intervention and non-intervention group seemed to increase over time (Figure 4) and differences seemed to become larger after the second measurement, which marked the start of MNP distribution. In contrast, the differences in WHZ between intervention and non-intervention seemed to decrease over time, while there was no change in differences in WAZ between the groups over time (Figure 4C,D).

The prevalence of stunting, wasting and underweight increased over time in both groups (Figure 2). However, a significantly smaller increase in stunting was observed in the intervention compared to the non-intervention group (+17.7% vs. +29.0%, OR for stunting at end line = 0.49; 95% CI: 0.40–0.60). A similar result was observed for underweight (+3.2% vs. +6.1%; OR for underweight at end line: 0.61; 95% CI: 0.47–0.79), whereas the change in wasting was not different between groups (OR at end line: 0.75; 95% CI: 0.50–1.11).

## 4. Discussion

The findings of this study show that even low iron dose MNP, provided every other day for eight months, can marginally improve haemoglobin concentrations and result in a remarkable improvement in linear growth when provided in the context of a CF program intervention. However, the low iron dose provided in this supplement may not have been sufficient to increase iron stores. MNP also resulted in increased diarrhoea and common cold/flu morbidity in the intervention compared to the non-intervention children. However, there were no differences in clinic visits as a proxy for severe disease, and the difference in longitudinal prevalence of diarrhoea became smaller over time, suggesting that the increased morbidity was most likely mild, and indications of a side effect upon introduction of the MNP.

The 11% non-significant reduction in prevalence of anaemia observed in our study is smaller than the 34% reduction shown in most other MNP studies [5,26]. In addition, contrary to our findings, most other studies found an effect on iron status as well. Compared to randomized controlled trials, program effectiveness studies in uncontrolled settings often result in lower adherence and subsequently smaller effect sizes [27]. The smaller observed effect on anaemia and no effect on iron status in our study may have been due to the lower than expected baseline anaemia prevalence in this age-group (36% anaemia observed at baseline in the intervention group vs. 73% expected based on the 2012 Ethiopia Demographic and Health Survey among 6–11-month-old children). In addition, the relatively low iron dose (6 mg/every 2 days) we provided may have been just enough for a small increase in haemoglobin levels, but not enough to fill iron stores, while the reduced growth retardation observed in the intervention children in our study suggest that the iron might have been used up by the body for growth, thus unavailable for storage. Contrary to expectations, we observed higher SF concentrations, a higher increase in SF and a lower prevalence of ID in the non-intervention children, which may be partly explained by the higher presence of acute inflammation, as shown by higher CRP and AGP levels at baseline in non-intervention children. While we corrected SF concentrations for inflammation by using the BRINDA approach [22], it is possible that this still did not remove all effects of the acute phase response on SF in this population. Even low levels of inflammation can reduce iron absorption due to elevated Hepcidin concentrations which was one of the explanations for the relatively low efficacy of MNP on iron status observed in a recent study in Kenya [28].

In our study, children receiving MNP supplementation were 2.3 times as likely as non-intervention children to develop diarrhoea and 2.1 times more likely to develop common cold and flu, but there was no effect on fever. Evidence of increased diarrhoea after MNP supplementation was also observed in a recent meta-analysis [26], although this effect was mainly based on the significant increase in diarrhoea observed in one large study in Pakistan [6], while no increase in morbidity was found in two smaller studies in Nepal [29] and Bangladesh [13]. Our study is comparable in sample size with the study in Pakistan, but we observed a lower overall diarrhoea morbidity in our study population (2.7% and 1.5% in intervention and non-intervention group) compared to the study in Pakistan (6.7% and 5.7%) [6]. Differences in morbidity between geographical locations may have been due to context-specific conditions, whereas the lower iron dose used in our study (6 mg iron every 2 days) as compared to the study in Pakistan (12.5 mg/day) may have contributed to the lower observed morbidity in the intervention group of our study. We did not find a difference in clinic visits between intervention and non-intervention group, which was used as a proxy for severe disease, and the difference in longitudinal prevalence became smaller over time, suggesting that the observed increase in morbidity was likely related to a mild disease, and indications of a side effect upon introduction of the MNP.

The increase in linear growth and weight gain levels observed in our study are remarkable, and the effect sizes are consistent with a limited number of other studies that show a significant effect of MNP on growth when provided combined with other nutrition or hygiene education interventions [13,30]. In contrast, MNP interventions alone are not likely to improve growth in most settings [5]. In our study as well, the improved growth cannot be fully attributed to the low dose iron MNP alone as the MNP provision was embedded in a program where locally produced CF was distributed. There is a growing level of evidence that combined MNP and IYCF interventions can prompt care-givers to improve complementary feeding practices [31,32]. The findings of our study suggest that, in an area with high levels of childhood malnutrition, such combined MNP and IYCF interventions may have the potential to dramatically reduce stunting levels, although the 51% reduction in stunting observed in our study is substantially larger than what has been observed in other settings. 

Our study had several limitations. The data collectors were not blinded to the intervention and this may have caused information bias. Although this may have affected the sizes of our effect estimates on morbidity and stunting somewhat, this would not change our overall conclusions. The study had a quasi-experimental design, in which intervention and non-intervention villages were not randomly assigned but purposely selected by the program implementers in close consultation with regional, zonal and district health bureaus. This could have created bias, particularly since intervention program villages were selected based on being more vulnerable and more in need of a community-based nutrition intervention. The differences in nutritional status observed at baseline seem to confirm this. Although non-intervention villages were matched with intervention villages based on socio-economic and demographic characteristics, differences between intervention and non-intervention villages in nutritional status and other key characteristics at baseline could therefore not be ruled out, even though we controlled for several of these differences in the analyses. Second, the lower than expected baseline prevalence of anaemia likely affected the power of our study to demonstrate differences in iron status. Thirdly, our analyses in the subsample for the biochemical analyses are further complicated by strong regional differences in, amongst others, dietary habits. While we matched the intervention and non-intervention group at district level resulting in an equal proportion in both regions, the sub-sample for biochemical assessment was selected by going from study village to village, until the required number was obtained. As a result, all children in the non-intervention group were from SNNPR, whereas most of the children (except 15) in the intervention group were from Oromia. Regional differences in dietary habits and a higher prevalence of food insecurity in Oromia during the time of this study likely contributed to the observed difference in iron status among children even at baseline [33]. For example, the major staple food in SNNP is *kocho*, which is known to be relatively rich in iron [34]. In contrast, the main staple food in Oromia is maize, which is known to be high in phytate, which affects iron absorption [34].

Strengths of our study are the implementation of the study in the context of a large-scale program-setting with eight-month duration, the longitudinal design and inclusion of matched-control villages in the design, and the large frequency of data collection involving a large number of children, providing adequate power to study differences in morbidity between groups. Finally, it is the first study providing evidence on the effectiveness of a low iron dose MNP-IYCN program on haemoglobin concentrations and linear growth.

## 5. Conclusions

MNP with low iron dose, when provided combined with other IYCF interventions, marginally improved haemoglobin status, without improving iron stores, and resulted in a remarkable improvement in linear growth in 6–23-month-old children, when provided in the context of a CF program intervention. These benefits likely outweigh the relative small increase in risk of diarrhoea, which seemed to be mostly mild in nature and disappeared over time. Nevertheless, programs introducing MNP in the context of an integrated IYCN intervention should ensure adequate management, monitoring and control of diarrhoea (with Oral Rehydration Solution (ORS) and zinc treatment).

## Figures and Tables

**Table 1:** Minerals and vitamins contents of MixMe per serving (1 g sachet).

Nutrient	Per 1 g
Vitamin A	1332 IU/400 mcg
Vitamin D	200 IU/5 mcg
Vitamin E	5 mg TE
Vitamin B_1_	0.5 mg
Vitamin B_2_	0.5 mg
Vitamin B_6_	0.5 mg
Vitamin B_12_	0.9 mcg
Niacin amide	6 mg
Folate	150 mcg
Vitamin C	30 mg
Iron	6 mg
Zinc	4.1 mg
Copper	0.56 mg
Selenium	17 mcg
Iodine	90 mcg

IU: International Unit; TE: Tocopherol Equivalent.

**Table 2:** Baseline characteristics of the study participants ^1^.

Characteristics		Intervention *n* = 1172		Non-Intervention *n* = 1137
Region, Oromia (%)	50.1		49.3	
**Child characteristics**				
Gender, Female (%)	49.4		47.5	
Age (mo)	7.9	(1.8)	8.1	(1.9) *
Hb (g/L) ^2^	112.4	(12.6)	115.0	(9.7) **
SF (µg/L) ^3^	13.9	(6.6,24.1)	14.0	(8.9,23.7) **
sTfR (mg/L) ^4^	6.0	(5.0,7.9)	5.4	(4.7,6.8) **
AGP(g/L) ^5^	0.9	(0.7,1.2)	1.2	(0.9,1.6)
CRP (mg/L) ^5^	1.5	(0.6, 4.8)	2.5	(0.9,6.0)
**Mother’s characteristics** ^6^				
Mother age (y)	25.3	(5.8)	25.7	(5.7)
Education, Illiterate (%)	49.9		44.8	*
**Household characteristics**				
Toilet facility—Pit latrine (%)	95.7		90.1	**
Access to safe drinking water (%) ^7^	93.2		92.3	
Land ownership ^8^ (%)	90.8		91.1	

^1^ Values are mean (SD), per cent, or median (25th and 75th percentiles). ^2^ Altitude adjusted, *n* = 129 intervention, *n* = 129 non-intervention. ^3^ Adjusted for inflammation using the Biomarkers Reflecting Inflammation and Nutritional Determinants of Anemia (BRINDA) Internal Regression Correction (Namaste et al., 2017). *n* = 109 intervention, *n* = 120 non-intervention. ^4^ *n* = 101 intervention, *n* = 118 non-intervention. ^5^ *n* = 109 intervention, *n* = 120 non-intervention. ^6^ *n* = 1171 for intervention, *n* = 1136 for non-intervention group. ^7^ Safe drinking water includes piped water (public tap and private tap), protected spring, protected well, water from borehole (in the yard and public), water from truck and rainwater [24]. ^8^ Although theoretically in Ethiopia land ownership always lies with the government, most families do have their own farming land to plough and produce agricultural produce. * *p* < 0.05, ** *p* < 0.001 difference between intervention and non-intervention tested with *t*-test for normally distributed variables, Mann–Whitney *U* test for not normally distributed variables, Chi-square for categorical variables.

**Table 3:** Change in iron status during the intervention period ^1^.

	Intervention		Non-Intervention		β(SE) ^2^		*p-*Value
**Hb (g/L) ^3^**							
Baseline	112.4	(12.6)	115.0	(9.7)			
End line	114.8	(10.5)	114.2	(8.7)			
Change ^4^	2.4	(1.17)	−0.8	(1.17)	3.17	(1.65)	0.056
**SF(ug/L) ^5^**							
Baseline	13.9	(6.6,24.1)	14.0	(8.9,23.7)			
End line	11.1	(5.8,22.6)	19.1	(11.0,35.6)			
Change ^4^	−2.11	(1.6)	6.4	(1.5)	−8.53	(2.14)	<0.0001
**sTfR (mg/L) ^6^**							
Baseline	6.0	(5.0,7.9)	5.4	(4.7,6.8)			
End line	5.0	(4.1,6.2)	4.2	(3.7,4.7)			
Change ^4^	−1.5	(0.4)	−1.6	(0.3)	0.11	(0.49)	0.820

^1^ Values are mean (SE) or median (25th and 75th percentiles); change is calculated as end line minus baseline; Hb, Haemoglobin (altitude adjusted); SF, serum ferritin (adjusted for inflammation using the Biomarkers Reflecting Inflammation and Nutritional Determinants of Anemia (BRINDA) Internal Regression Correction (Namaste et al., 2017)); sTfR, serum transferrin receptor. ^2^ Regression coefficient (SE) comparing intervention with non-intervention group in Generalized Linear Model (GLM) analysis with change in Hb, SF, and sTfR as dependent variable; gender and treatment group as fixed factors; and age at baseline as covariate. ^3^ *n* = 129 for intervention, *n* = 129 for non-intervention. ^4^ Change is calculated as end line minus baseline. ^5^ *n* = 108 for intervention, *n* = 118 for non-intervention. ^6^ *n* = 101 for intervention, *n* = 118 for non-intervention.

**Table 4:** Prevalence and incidence of diarrhoea, common cold and flu, and fever during the intervention period.

Variable	Intervention (*n* = 1148)	Non-Intervention (*n* = 1125)	
**Longitudinal Prevalence (%) ^2^**			**β (95% CI) ^1^**
Diarrhoea	2.7 (3.6)	1.5 (3.2)	1.01 (0.73,1.29) *
Common cold and flu	5.4 (5.4)	2.7 (3.7)	2.44 (2.08,2.80) *
Fever	0.1 (0.2)	0.1 (0.3)	−0.01 (−0.03,0.01)
**Incidence Rate (per child/ year) ^4^**			**IRR (95% CI) ^3^**
Number of observation days	204,456	210,686	
Diarrhoea	2.67 (1474)	1.34 (786)	2.31 (1.92,2.78) *
Common cold and flu	3.77 (2178)	1.90 (1109)	1.43 (1.23,1.65) *
Clinic visits due to diarrhoea (per year) ^5^	0.41 (470)	0.37 (415)	1.23 (0.86,1.77)
Clinic visits due to common cold and flu (per year) ^5^	0.30 (349)	0.38 (431)	0.90 (0.62,1.32)

^1^ Regression coefficient expressing difference in longitudinal prevalence (i.e., per cent of days sick out of total number of observation days) between intervention and non-intervention groups from Generalized Linear Mixed Models (GLMM) using age, gender, pre-intervention outcomes and matching pairs as covariates. ^2^ Values are mean percentage (SD). ^3^ IRR, Incidence Rate Ratio; 95% CI, 95% Confidence Interval, from Poisson regression using number of episodes as dependent variable, pre-intervention morbidity cases (diarrhoea and flu case), age, gender, and matching pairs as covariates. IRRs of clinic visits were analysed with Poisson regression using number of clinic visits as dependent variable and age, gender and matching pairs as covariates. ^4^ Mean incidence/child/year (total number of episodes) for incidence rate. ^5^ Average number of clinic visits per child per year (total number of clinic visits for the group). * *p* < 0.001.

**Table 5:** Growth status of children during the intervention period ^1.^

	Intervention		Non-Intervention		β(SE) ^2^	
**Height, cm**						
Baseline	68.4	(3.8)	67.9	(4.0)		
1st Quarter	73.6	(3.6)	72.7	(3.9)	0.55	(0.09) **
2nd Quarter	77.2	(3.8)	76.1	(3.9)	0.67	(0.11) **
End line	80.0	(3.9)	78.8	(4.0)	0.77	(0.13) **
**Weight, kg**						
Baseline	7.8	(1.1)	7.6	(1.1)		
1st Quarter	8.9	(1.2)	8.6	(1.2)	0.15	(0.03) **
2nd Quarter	9.6	(1.2)	9.3	(1.2)	0.13	(0.04) **
End line	10.2	(1.3)	9.9	(1.3)	0.08	(0.04) *
**HAZ**						
Baseline	−0.49	(1.37)	−0.80	(1.43)		
1st Quarter	−1.12	(1.26)	−1.53	(1.35)	0.10	(0.04) *
2nd Quarter	−1.31	(1.24)	−1.80	(1.29)	0.18	(0.04) **
End line	−1.43	(1.22)	−1.91	(1.27)	0.18	(0.05) **
**WHZ**						
Baseline	−0.27	(1.08)	−0.42	(1.13)		
1st Quarter	−0.22	(1.06)	−0.43	(1.14)	0.04	(0.04)
2nd Quarter	−0.19	(1.00)	−0.33	(1.02)	−0.02	(0,04)
End line	−0.24	(1.03)	−0.31	(1.14)	−0.09	(0.05)
**WAZ**						
Baseline	−0.54	(1.27)	−0.83	(1.15)		
1st Quarter	−0.70	(1.11)	−1.08	(1.15)	0.07	(0.03) *
2nd Quarter	−0.76	(1.06)	−1.11	(1.06)	0.06	(0.04)
End line	−0.88	(1.05)	−1.18	(1.10)	0.01	(0.04)
**HAD**						
Baseline	−1.11	(3.13)	−1.81	(3.28)		
1st Quarter	−2.84	(3.22)	−3.89	(3.42)	0.33	(0.09) **
2nd Quarter	−3.61	(3.41)	−4.97	(3.55)	0.66	(0.11) **
End line	−4.20	(3.61)	−5.65	(3.71)	0.78	(0.12) **

^1^ Values are mean (SD) unless stated otherwise. HAZ, Height for Age *z*-score; WHZ, Weight for Height Z-score; WAZ, Weight for Age *z*-score; HAD, Height for Age difference based on 2006 World Health Organsiation (WHO) reference population [25]. ^2^ Regression coefficient (SE) for interaction between time and treatment group with baseline as reference from Linear Mixed Models (LMM) of growth status adjusting for age at baseline, gender, and matching pairs. * *p* < 0.05, ** *p* < 0.001.
